# Impact of surgical pulmonary valve replacement on ventricular strain and synchrony in patients with repaired tetralogy of Fallot: a cardiovascular magnetic resonance feature tracking study

**DOI:** 10.1186/s12968-018-0460-0

**Published:** 2018-06-18

**Authors:** Sowmya Balasubramanian, David M. Harrild, Basavaraj Kerur, Edward Marcus, Pedro del Nido, Tal Geva, Andrew J. Powell

**Affiliations:** 1 Department of Cardiology, Boston Children’s Hospital, Boston, USA; 2 Department of Pediatrics, Harvard Medical School, Boston, USA; 3 Department of Cardiac Surgery, Boston Children’s Hospital, Boston, USA; 4 Department of Surgery, Boston Children’s Hospital, Boston, USA

**Keywords:** Tetralogy of fallot, Pulmonary valve replacement, Feature tracking, Myocardial strain, Ventricular synchrony

## Abstract

**Background:**

In patients with repaired tetralogy of Fallot (TOF), a better understanding of the impact of surgical pulmonary valve replacement (PVR) on ventricular mechanics may lead to improved indications and outcomes. Therefore, we used cardiovascular magnetic resonance (CMR) feature tracking analysis to quantify ventricular strain and synchrony in repaired TOF patients before and after PVR.

**Methods:**

Thirty-six repaired TOF patients (median age 22.4 years) prospectively underwent CMR a mean of 4.5 ± 3.8 months before PVR surgery and 7.3 ± 2.1 months after PVR surgery. Feature tracking analysis on cine steady-state free precession images was used to measure right ventricular (RV) and left ventricular (LV) circumferential strain from short-axis views at basal, mid-ventricular, and apical levels; and longitudinal strain from 4-chamber views. Intraventricular synchrony was quantified using the maximum difference in time-to-peak strain, the standard deviation of the time-to-peak, and cross correlation delay (CCD) metrics; interventricular synchrony was assessed using the CCD metric.

**Results:**

Following PVR, RV end-diastolic volume, end-systolic volume, and ejection fraction declined, and LV end-diastolic volume and end-systolic volume both increased with no significant change in the LV ejection fraction. LV global basal and apical circumferential strains, and basal synchrony improved. RV global circumferential and longitudinal strains were unchanged, and there was a varied impact on synchrony across the locations. Interventricular synchrony worsened at the midventricular level but was unchanged at the base and apex, and on 4-chamber views.

**Conclusions:**

Surgical PVR in repaired TOF patients led to improved LV global strain and no change in RV global strain. LV and RV synchrony parameters improved or were unchanged, and interventricular synchrony worsened at the midventricular level.

## Background

Young children undergoing surgical repair of tetralogy of Fallot (TOF) have excellent short-term survival [1]; however, they experience significant morbidity and mortality related to biventricular dysfunction and arrhythmia in their adult years [2–4]. These sequelae are believed to be in part related to chronic pulmonary regurgitation (PR) caused by efforts to relieve pulmonary valve stenosis with the initial repair. Thus, surgical pulmonary valve replacement (PVR) is often performed subsequently to improve the long-term outcome [5, 6]. Although a reduction in PR, right ventricular (RV) end-diastolic volume (EDV), and RV end-systolic volume (ESV) is observed, the beneficial impact of PVR on ventricular systolic function, exercise capacity, arrhythmia, and survival is uncertain [6–9].

A better understanding of the effects of surgical PVR on ventricular function may lead to improved indications and outcomes for the procedure. In particular, areas that merit further investigation are regional ventricular mechanics and synchrony. In repaired TOF patients, right bundle branch block is nearly universal and several studies have documented dyssynchronous ventricular contraction [10–12]. Moreover, left ventricular (LV) dysfunction is seen in conjunction with RV dysfunction suggesting adverse ventricular-ventricular interactions [3, 13, 14]. Nevertheless, our knowledge of regional ventricular mechanics in this patient group remains rudimentary, in part because robust techniques for its assessment have not been applied.

Feature tracking is an image processing technology that quantifies myocardial tissue deformation, and has been increasingly employed to aid cardiac resynchronization therapy [15, 16]. This technique has primarily been applied to ultrasound images; however, recent work has adapted and validated its use with cardiovascular magnetic resonance (CMR) images in both the right and left ventricles [12, 17–21]. CMR offers the advantage over echocardiography of consistent high-quality imaging of both ventricles in patients with repaired TOF.

Our group previously published a prospective, randomized study comparing 2 techniques for surgical PVR: PVR alone versus PVR plus RV remodeling with the resection of scar tissue [22]. No significant difference was observed in the primary outcome−change in RV ejection fraction (EF) measured by CMR−or in any of the secondary outcomes at 6-month postoperative follow-up. In this study, we performed feature tracking image analysis on data from the trial cohort to determine the impact of surgical PVR on strain and ventricular synchrony in patients with repaired TOF.

## Methods

### Subjects

Subjects in this study were all participants in a previously reported prospective, randomized, single-center trial comparing 2 techniques for surgical PVR [22]. These patients were > 10 years of age with repaired TOF or similar physiology presenting for PVR between February 2004 and October 2008. Other inclusion criteria in the prior study included chronic PR (regurgitation fraction by CMR ≥25%) and at least 2 of the following conditions: RV EDV index ≥160 ml/m^2^, RV ESV index ≥70 ml/m^2^, RV EF ≤45%, LV EDV index ≤65 ml/m^2^, RV outflow aneurysm, or a clinical criterion such as exercise intolerance, symptoms and signs of heart failure, or cardiac medications. Exclusion criteria included any of the following: severe RV outflow obstruction, severe RV hypertension with RV pressure ≥ systemic pressure, and contraindications to preoperative CMR. Patients were randomized to PVR alone or a combination of PVR with RV remodeling surgery. The study protocol included a pre-operative and a 6-month post-operative CMR study.

For the current study, we analyzed the database of the prospective trial, selected only those patients with TOF, and performed feature tracking analysis of the CMR images. The Boston Children’s Hospital Committee on Clinical Investigation granted permission for this study and waived the requirement for informed consent.

### CMR

The CMR protocol used for patients with repaired TOF has been previously published [23]. Briefly, studies were performed with a 1.5-T whole body scanner (Achieva, Phillips Healthcare Systems, Best, the Netherlands or TwinSpeed, GE Healthcare, Milwaukee, Wisconsin, USA) using surface coils selected based on patient size. The scanner manufacturer was different for the pre- and post-PVR CMR study in 4 patients. Imaging included breath-hold, electrocardiographically-gated, balanced steady-state free precession cine CMR acquisitions in 4-chamber and short-axis planes. A total of 12−14 slices were obtained in the short-axis plane to completely cover both ventricles. In all views, 30 images per cardiac cycle were acquired which yielded a temporal resolution of 20–40 ms, depending on heart rate. Ventricular volumes and blood flow were measured using commercially available software (QMASS and QFLOW, Medis, Leiden, the Netherlands) [23].

### Strain analysis

**Fig. 1:**
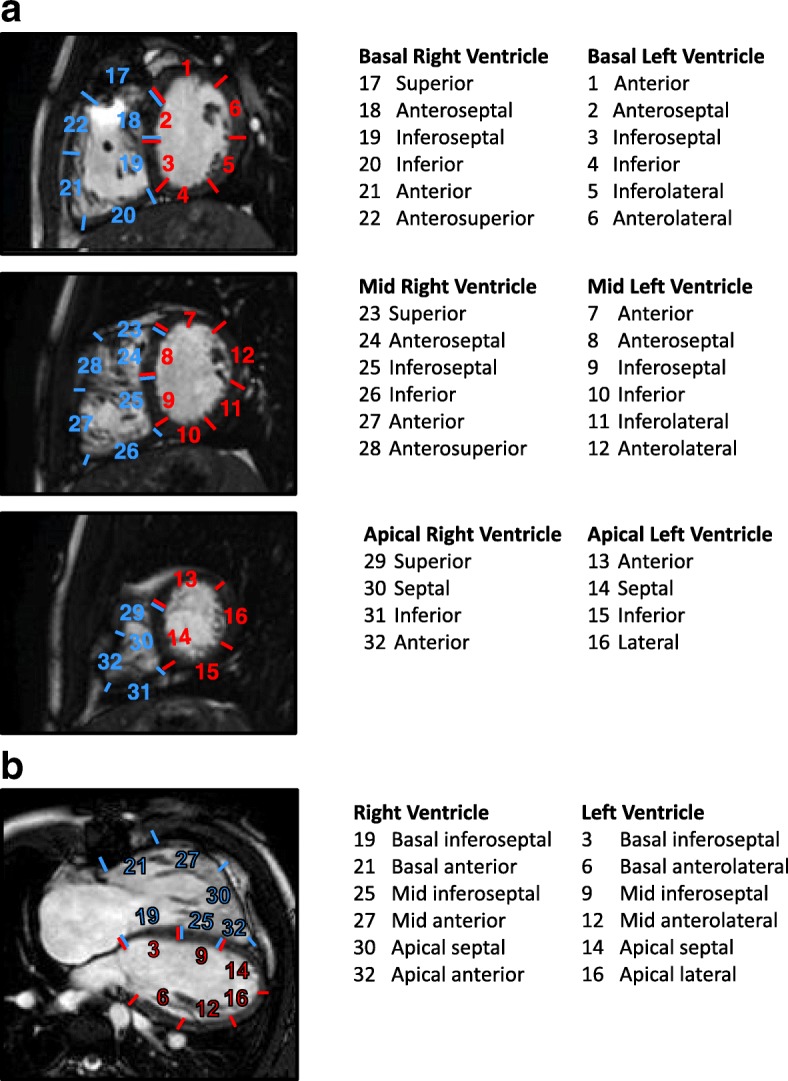
Schematic diagram illustrating the division of the right ventricle (RV) and left ventricle (LV) myocardium into segments on short-axis (**a**) and 4-chamber views (**b**)

Feature tracking analysis for determination of strain was performed using commercially available software (Diogenes v. 1.1.02, TomTec Imaging Systems, Unterschleissheim, Germany), as previously described [12]. Circumferential strains for the LV and RV were measured from short-axis views at 3 levels: basal, mid-ventricular, and apical. The mid-ventricular level was first identified, and the basal and apical slices were specified to be equidistant from the mid-ventricular slice. The LV outflow tract was not included in the basal slice. Pre- and post-PVR images on each subject were compared to ensure similar slice locations. For short-axis views, the myocardium of each ventricle was divided equally into 6 segments at the mid and basal locations, and 4 segments at the apical location (Fig. 1a). Longitudinal strains for the LV and RV were measured from the 4-chamber view with division into 6 segments (Fig. 1b). Global peak strain for each view was calculated as the average of the peak strains for each segmental curve [17, 24]. Strain measurements were performed by the same investigator in all patients in order to promote consistency. In addition, they were repeated by that investigator and performed by a second investigator in a random sample of patients to assess intraobserver and interobserver agreement. Note that strain is calculated as follows: (final length – initial length) / initial length. Thus, shortening in the longitudinal and circumferential directions yields a negative strain number, and that the more negative the number, the better the shortening.

### Synchrony analysis

Various ventricular synchrony parameters based on feature tracking measurements have been reported; however, there is no consensus on the optimal approach. Hence, we used 3 techniques that have been reported in the literature as measures of ventricular synchrony [17, 25, 26]. Three ventricular synchrony parameters were calculated for each ventricle on each of the 3 short-axis views and the 4-chamber view based on the strain versus time data: 1) the maximum difference in time-to-peak strain among any 2 of the segments (latest versus earliest segment), 2) the standard deviation of the time-to-peak strain values for all segments, and 3) the cross-correlation delay (CCD). The time to peak strain was measured from the QRS trigger to the peak strain time. Using a custom built virtual instrument (LabVIEW 8.2, National Instruments, Austin, Texas, USA), the CCD was measured by iteratively shifting 1 curve in time relative to a second curve in a stepwise fashion, and calculating the normalized correlation coefficient between the curves for each time shift [25, 27]. The time shift that resulted in the maximum correlation coefficient was defined as the CCD between the 2 curves. Rather than relying on just the peak points, CCD takes into account the entire strain versus time curve to determine the temporal offset. For each ventricle on each of the 4 views, the CCDs were measured between opposing segments, and the largest value of the opposing segment CCDs was reported. In addition, the interventricular CCD on each of the 4 views was calculated by comparing the global strain versus time curves for the LV and RV.

### Statistical analysis

All collected data were tested for normalcy using the Shapiro-Wilk test. Continuous data are presented as median (range) and mean ± standard deviation. A two-tailed paired t-test was used to compare pre- and post-PVR strain and synchrony parameters. A *p*-value ≤0.05 was considered statistically significant. The intraclass correlation coefficient (ICC) and mean difference were used to assess intraobserver and interobserver agreement.

## Results

### Patients

Of the 64 patients in the initial prospective trial, 36 had a diagnosis of TOF as well as pre- and post-operative CMR studies with suitable image quality for strain analysis. These patients constitute the study group, and their demographic and clinical data are summarized in Table 1. Thirteen patients were randomized to the PVR alone group and 23 to the PVR with RV remodeling surgery group. CMR examinations occurred at a mean of 4.5 ± 3.8 (range 0.03–12.7) months before PVR surgery and 7.3 ± 2.1 (range 5.2–10.8) months after surgery.

**Table 1:** Patient characteristics (*n* = 36)

Male	23 (64%)
Prior aortopulmonary shunt	10 (28%)
Age at initial complete repair (years)	1.5 (0.0–19.4)
Age at PVR (years)	22.4 (12.3–57.7)
Cardiac symptoms prior to PVR	21 (58%)
NYHA Class I or II prior to PVR	33 (92%)
NYHA Class III or IV prior to PVR	3 (8%)
Prescribed cardiac medications prior to PVR	16 (44%)
Additional procedures with PVR	
Closure of patent foramen ovale or atrial septal defect	8 (22%)
Pulmonary artery plasty	10 (28%)
Tricuspid annuloplasty	6 (17%)
Cryoablation	2 (6%)
Maze procedure	4 (11%)
Closure of ventricular septal defect	3 (8%)
Other	4 (11%)

Data presented as n (%) or median (range)

*PVR* pulmonary valve replacement

### Diagnostic testing results

Key diagnostic testing results pre- and post-PVR are shown in Table 2. As expected, PVR led to virtual elimination of PR, and a significant decline in RV EDV and ESV. These changes yielded a slight decline in RV EF. The LV EDV and ESV both increased slightly with no significant change in the LV EF. There was no significant change in QRS duration or the heart rate at the time of CMR.

**Table 2:** Diagnostic testing results pre- and post-PVR (*n* = 36)

Parameter	Pre-PVR	Post-PVR	*P*-value
Electrocardiogram			
QRS duration (ms)	153 ± 38	152 ± 39	0.99
Exercise Testing			
Peak VO_2_ (ml/kg/min)	26 ± 9	25 ± 8	0.31
Echocardiography			
≤ Mild tricuspid regurgitation	33	35	
≥ Moderate tricuspid regurgitation	3	1	
RV systolic pressure by tricuspid regurgitation jet velocity	35 ± 15	25 ± 7	0.009
CMR			
Heart rate	75 ± 11	73 ± 13	0.38
LV EDV_i_ (ml/m^2^)	88 ± 17	93 ± 17	0.01
LV ESV_i_ (ml/m^2^)	37 ± 10	40 ± 12	0.02
LV ejection fraction (%)	58 ± 8	57 ± 7	0.35
RV EDV_i_ (ml/m^2^)	194 ± 34	119 ± 16	< 0.001
RV ESV_i_ (ml/m^2^)	100 ± 24	65 ± 16	< 0.001
RV ejection fraction (%)	49 ± 7	46 ± 7	0.02
Pulmonary regurgitation fraction (%)	48 ± 10	4 ± 5	< 0.0001

*EDV* end-diastolic volume, *ESV* end-systolic volume, *LV* left ventricular, *PVR* pulmonary valve replacement, *RV* right ventricular

### Strain analysis

**Fig. 2:**
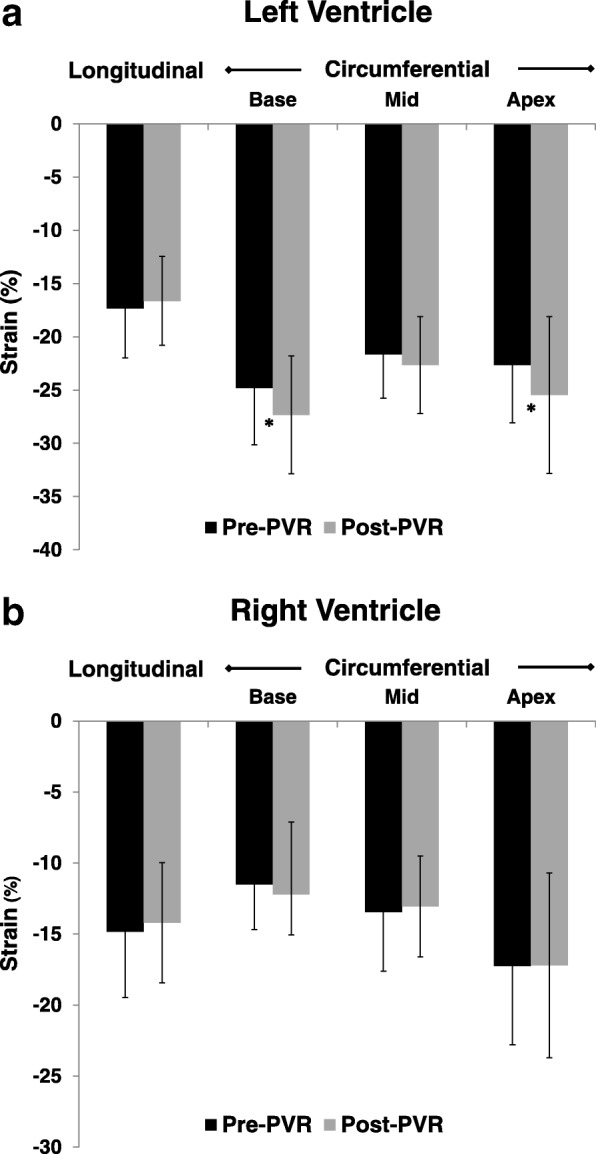
Global longitudinal and circumferential strain in the LV (**a**) and RV (**b**) pre- and post-pulmonic valve replacement (PVR). An asterisk (*) indicates a statistically significant change. *LV – left ventricle, RV – right ventricle, PVR – pulmonary valve replacement*

Global strain values pre- and post-PVR are shown in Fig. 2. In the LV, global circumferential strain at the base and apex improved following PVR; strain at the mid ventricle also tended to improve but this did not reach statistical significance. Global longitudinal strain was unchanged. In the RV, global circumferential strain at all 3 levels and global longitudinal strain were unchanged.

**Fig. 3:**
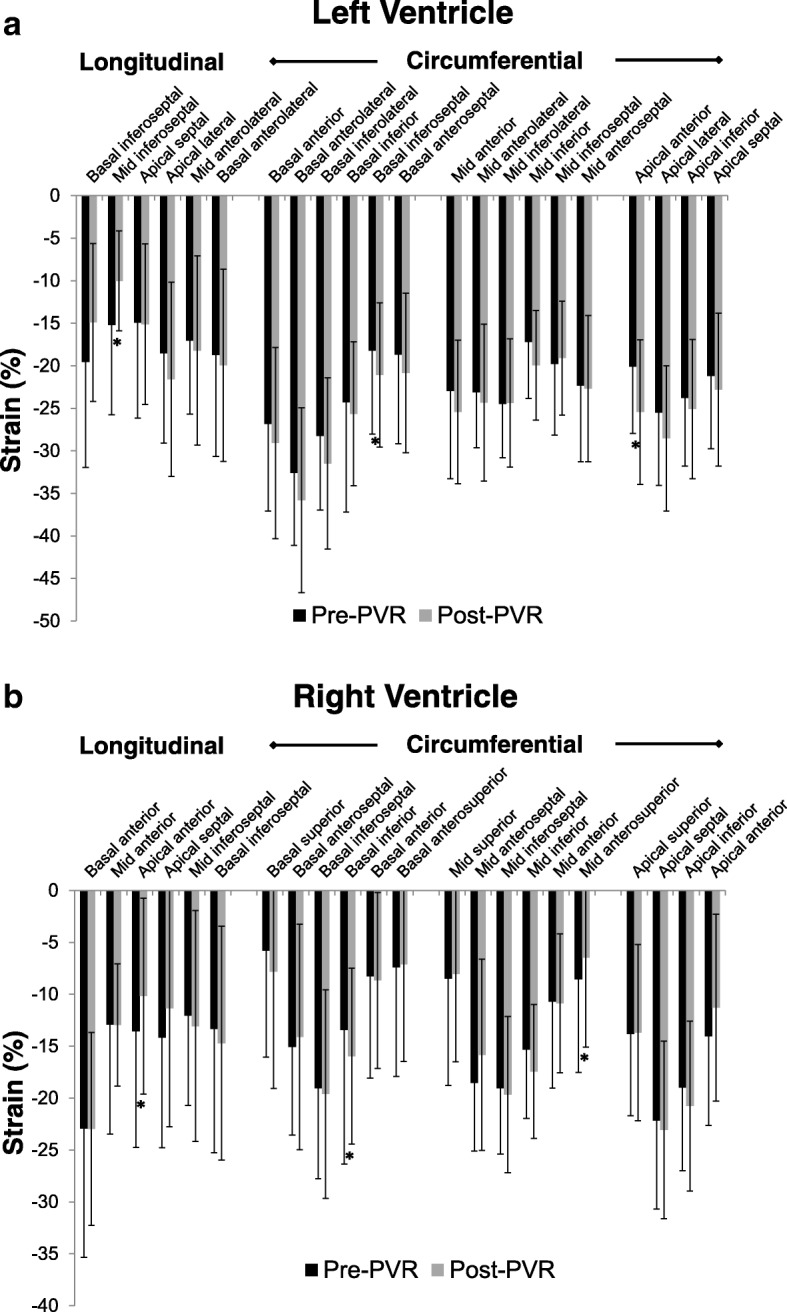
Segmental longitudinal and circumferential strain in the LV (**a**) and RV (**b**) pre- and post-PVR. An asterisk (*) indicates a statistically significant change. *LV – left ventricle, RV – right ventricle, PVR – pulmonary valve replacement*

The impact of PVR on segmental strain in the LV and RV is shown in Fig. 3. Overall, changes in regional stain were minimal. Significant differences in circumferential LV strain were seen in 2 of 16 segments, and these were both improvements. Longitudinal strain changed in 1 of 6 segments and this was a decline. Significant differences in circumferential RV strain were seen in 2 of 16 segments, 1 increase and 1 decrease. Longitudinal strain changed in 1 of 6 segments and this was a decline.

### Synchrony analysis

Synchrony parameters derived from the segmental strain versus time curves pre- and post-PVR are shown in Table 3. For LV circumferential strain, all 3 parameters showed significantly improved synchrony post-PVR at the base. At the mid and apex levels, all parameters tended to improve and some met the significance threshold. There were no significant synchrony changes based on longitudinal strain.

**Table 3:** Synchrony parameters pre- and post-PVR (*n* = 36)

Parameter	Pre-PVR	Post-PVR	*P*-value
Left ventricle			
Longitudinal strain			
Maximum difference in time-to-peak	315 ± 185	351 ± 196	0.33
Standard deviation of time-to-peak	121 ± 73	139 ± 82	0.23
Cross-correlation delay	301 ± 218	340 ± 242	0.39
Circumferential strain: base			
Maximum difference in time-to-peak	233 ± 172	151 ± 120	0.01
Standard deviation of time-to-peak	89 ± 63	58 ± 48	0.02
Cross-correlation delay	217 ± 182	107 ± 77	0.0001
Circumferential strain: mid			
Maximum difference in time-to-peak	124 ± 75	107 ± 55	0.02
Standard deviation of time-to-peak	49 ± 27	43 ± 20	0.15
Cross-correlation delay	106 ± 113	77 ± 53	0.13
Circumferential strain: apex			
Maximum difference in time-to-peak	58 ± 46	47 ± 44	0.27
Standard deviation of time-to-peak	28 ± 22	23 ± 21	0.26
Cross-correlation delay	99 ± 146	42 ± 24	0.04
Right ventricle			
Longitudinal strain			
Maximum difference in time-to-peak	249 ± 176	337 ± 170	0.02
Standard deviation of time-to-peak	97 ± 70	128 ± 64	0.02
Cross-correlation delay	258 ± 171	254 ± 179	0.91
Circumferential strain: base			
Maximum difference in time-to-peak	361 ± 198	265 ± 155	0.01
Standard deviation of time-to-peak	136 ± 70	99 ± 59	0.004
Cross-correlation delay	342 ± 285	341 ± 286	0.56
Circumferential strain: mid			
Maximum difference in time-to-peak	214 ± 133	268 ± 179	0.11
Standard deviation of time-to-peak	96 ± 55	101 ± 66	0.73
Cross-correlation delay	268 ± 216	263 ± 224	0.93
Circumferential strain: apex			
Maximum difference in time-to-peak	95 ± 84	129 ± 97	0.047
Standard deviation of time-to-peak	43 ± 37	60 ± 43	0.03
Cross-correlation delay	119 ± 148	126 ± 145	0.82

Values expressed as mean ± standard deviation in ms

For the RV, both of the time-to-peak parameters showed significantly improved synchrony at the base and worsened at the apex and longitudinally. At the mid location, there were no significant synchrony changes.

**Fig. 4:**
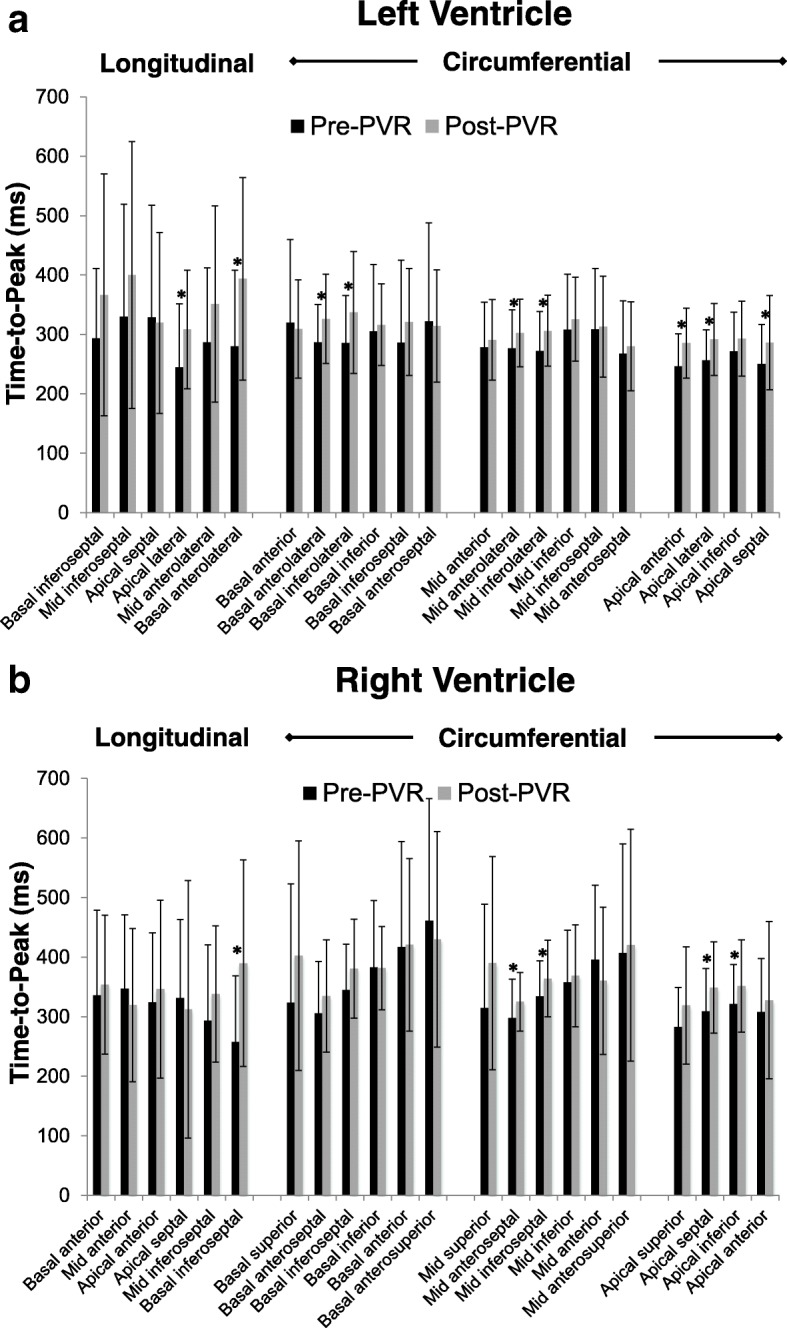
Segmental time-to-peak longitudinal and circumferential strain in the LV (**a**) and RV (**b**) pre- and post-PVR. An asterisk (*) indicates a statistically significant change. *LV – left ventricle, RV – right ventricle, PVR – pulmonary valve replacement*

In addition to the synchrony parameters reported above, the raw time-to-peak strain values (rather than differences) for each segment were compared pre- and post-PVR. This analysis provides information on how PVR affects the timing of contraction relative to the cardiac cycle rather than assessing synchrony per se. All LV segments with a statistically significant change in time-to-peak strain following PVR were increases and these were 2 of the 6 longitudinal and 7 of 16 circumferential segments (Fig. 4a). Similarly, all RV segments with a statistically significant change in time-to-peak strain after PVR were increases, and these were 1 of the 6 longitudinal and 4 of the 16 circumferential segments (Fig. 4b).

Interventricular synchrony was assessed by comparing the global strain versus time curves for the LV and RV on each of the 4 views, and calculating the CCDs. The LV peak strain preceded RV peak strain in 128 of 144 segments (89%) pre-PVR and in 127 of the 144 segments (88%) post PVR. The interventricular CCD significantly increased after PVR at the mid-ventricular level (50.4 ± 26.4 ms vs. 66.7 ± 29.1 ms, *p* = 0.002), as a result of slightly earlier LV peak strain. There was no change in the interventricular CCD at the base (76.5 ± 53.8 ms vs. 90.5 ± 56.2 ms, *p* = 0.62), the apex (46.8 ± 34.4 ms vs. 49.0 ± 33.2 ms, *p* = 0.73), or the 4-chamber view (61.8 ± 109.6 ms vs. 52.3 ± 86.7 ms, *p* = 0.69).

### Intraobserver and interobserver agreement

Table 4 shows the intraobserver and interobserver agreement results for global strain and synchrony parameters. Intraobserver agreement was good for LV circumferential and longitudinal strain, and for RV circumferential strain (all ICCs ≥0.93), and acceptable for RV longitudinal strain (ICC 0.85). Interobserver agreement for these parameters was lower. Compared to global strains, time-to-peak strain and CCD generally had lower ICCs; nevertheless, intraobserver ICCs for these LV synchrony parameters were all ≥0.73. Agreement for RV synchrony values derived from longitudinal strain curves was only fair.

**Table 4:** Interobserver and intraobserver variability for global strain and synchrony parameters

Parameter	Intraobserver (*n* = 10)		Interobserver (*n* = 10)	
	Mean difference ± SD	ICC	Mean difference ± SD	ICC
Left ventricle				
Circumferential – mid				
Global strain (%)	0.25 ± 1.3	0.96	0.30 ± 1.9	0.89
Maximum difference T2P (ms)	12.5 ± 27.8	0.86	1.0 ± 38.5	0.82
Cross-correlation delay (ms)	2.8 ± 21.9	0.83	14.6 ± 31.4	0.62
Longitudinal				
Global strain (%)	0.33 ± 2.1	0.93	1.6 ± 3.7	0.75
Maximum difference T2P (ms)	102.3 ± 145	0.80	40.0 ± 154	0.67
Cross-correlation delay (ms)	25.0 ± 80.7	0.73	45.8 ± 107	0.65
Right ventricle				
Circumferential – mid				
Global strain (%)	0.40 ± 1.6	0.95	0.31 ± 2.0	0.88
Maximum difference T2P (ms)	64.0 ± 126	0.82	90.6 ± 107.9	0.61
Cross-correlation delay (ms)	104 ± 233	0.66	93.0 ± 230	0.59
Longitudinal				
Global strain (%)	1.0 ± 2.8	0.85	2.6 ± 2.2	0.76
Maximum difference T2P (ms)	146 ± 133	0.76	157 ± 158	0.66
Cross-correlation delay (ms)	22.4 ± 85.9	0.64	42.1 ± 118	0.53

*ICC* intraclass correlation coefficient, *T2P* time-to-peak, *SD* standard deviation

## Discussion

This is the first study to use CMR to assess the impact of surgical PVR on myocardial strain and synchrony in patients with repaired TOF. At a mean of 7 months following PVR, LV global basal and apical strain, and basal synchrony improved. PVR had no impact on RV global circumferential and longitudinal strains, and a varied impact on synchrony across the locations. Interventricular synchrony worsened at the midventricular level but was unchanged at the base and apex, and on 4-chamber views. These results provide new insight into the effects of PVR on ventricular mechanics and may prompt modifications in management.

Based on multiple studies published over the past decade in repaired TOF patients, a clear picture has emerged regarding the short-term effects of surgical PVR on ventricular volumes and EF [5, 6, 28]. On average, RV EDV decreases by 30–40%, LV EDV increases slightly, and RV and LV EF largely remain unchanged. This response was also seen in our study cohort.

Analysis of these global parameters, however, is likely insufficient to fully characterize the effects of PVR on ventricular mechanics. Myocardial deformation indices are thought to be more sensitive markers of ventricular function than volumetric parameters [12, 13, 29]. Our finding of improved LV circumferential strain and synchrony following PVR in the absence of appreciable changes in LV EF follows this premise. The utility of echocardiography-based strain measurement techniques in repaired TOF patients is limited by diminished acoustic windows and visualization, particularly for the RV. CMR has the advantage of providing consistently high image quality for both the RV and LV myocardial walls. There are several CMR methods for myocardial deformation imaging; however, only feature tracking can be applied to standard cine images without performing additional time-consuming imaging sequences. The use of this approach for our study is further supported by reports from our group and others that have shown acceptable correlation between feature tracking and the more established myocardial tagging technique for strain measurement [18, 30, 31]. Investigations have also found that intraobserver and interobserver agreement for radial strain by CMR feature tracking is lower than that for circumferential and longitudinal strain [20, 30]. For this reason, radial strain assessment was not included in the current study. In our study, intraobserver agreement for LV and RV strain and LV synchrony parameters was good; for RV synchrony parameters, it was only fair. Interobserver agreement for several of the strain and synchrony parameters was rather low, which suggests a limited clinical role of this technique for assessing RV synchrony in particular. However, the high intraobserver reliability for most parameters is of primary importance in this study as all of the pre- and post-PVR measurements were made by a single observer.

As the optimal technique to assess ventricular synchrony is unknown, we examined the effect of PVR on 3 metrics extracted from segmental strain versus time curves—maximum difference in time-to-peak, standard deviation of time-to-peak, and CCD. In our analysis, the most robust change with PVR was a significant improvement in intraventricular RV and LV synchrony at the basal level. Interventricular synchrony did not change in 3 of the 4 views; the one statistically significant difference was a mild worsening of synchrony in the mid-ventricular view related to earlier LV contraction. QRS duration was unchanged.

Our data demonstrated for the most part concordant changes in strain and synchrony parameters in the LV supporting a causal relationship. Specifically, following PVR, most of the circumferential strain-based synchrony parameters significantly improved at the base and apex of the LV. This enhanced coordination of contraction may have contributed to the improvements in LV global circumferential strain that were also seen in the same segments. More favorable ventricular-ventricular interactions in diastole may also have contributed to the improved LV strain [32]. The near elimination of PR with PVR would be expected to reduce the pressure difference across the ventricular septum, and, therefore, diminish diastolic septal bowing toward the LV. The resultant improved LV filling increases preload and muscle fiber stretch leading to increased strain by the Frank–Starling mechanism. This explanation is supported by the increase in indexed LV EDV seen after PVR.

### Prior studies

Only a few other reports have measured changes in strain that accompany PVR. A similar study from our group examined the impact of transcatheter pulmonary valve implantation on strain assessed by CMR feature tracking [17]. All patients (*n* = 31) had RV-to-pulmonary artery conduit dysfunction, and were divided into those with predominant pulmonary stenosis (*n* = 18) and those with predominant PR (*n* = 13). For the PR subgroup, PVR led to not only improved LV circumferential strain, as it did in our patients with surgical PVR, but also improved longitudinal strain. This difference in longitudinal strain response may be related to the fact that even in the PR subgroup, there was some degree of pulmonary stenosis, more than in the current study population. There was no change in RV circumferential or longitudinal strain with transcatheter PVR in the PR subgroup, in agreement with our results for surgical PVR.

Prior studies assessing the impact of PVR on ventricular synchrony are also scarce. Lurz et al. examined 20 patients with RV-to-pulmonary artery conduit obstruction, most with TOF, before and after transcatheter PVR [14]. Based on tissue Doppler echocardiography from an apical 4-chamber view, the time-to-peak strain for the RV free wall decreased and for the LV free wall was unchanged, leading to significantly less interventricular mechanical delay following PVR. Again, the presence of RV outflow tract obstruction confounds the comparison to our results. However, similar to our study, the QRS duration was unchanged highlighting the importance of assessing both mechanical and electrical measures of ventricular synchrony.

### Clinical implications

The clinical implications of improved LV circumferential strain and synchrony following PVR are uncertain but there are some data to suggest that this may be beneficial. Impaired LV systolic function in repaired TOF patients is fairly common [33, 34]. Both reduced LV global function as well as deformation indices have been shown to be important independent predictors of clinical deterioration, arrhythmia, and death in adults after TOF repair [3, 4, 24, 29, 34–36]. Moreover, our group has shown that LV dyssynchrony is associated with ventricular tachycardia and death in repaired TOF patients [12]. It is thus possible that the improvements in these parameters seen with PVR may lead to a better prognosis, and further study is warranted. For the RV in TOF, global systolic dysfunction is common and associated with adverse outcomes [4, 24]. Abnormalities in RV deformational indices are also prevalent [10, 37, 38]. Our data showed no improvement in any of these parameters with PVR. This finding suggests that strategies other than PVR may need to be pursued to achieve meaningful changes in outcomes related to impaired RV function. To this end, our data supports efforts to maintain pulmonary valve function and optimize myocardial preservation at the time of initial repair as later PVR cannot be relied on to restore function. Similarly, antifibrotic pharmacologic therapy should be explored as studies have implicated fibrosis in the pathophysiology of myocardial dysfunction [39–41]. The lack of improvement in RV functional indices may be because restoration of normal loading conditions occurred after the myocardium had sustained irreversible damage. Accordingly, future studies with larger numbers should assess whether earlier PVR leads to a better RV response. Lastly, our data showed that PVR did not lead to a convincing improvement in interventricular synchrony. Thus, for repaired TOF patients in whom interventricular dyssynchrony is thought to be playing a clinically important role, cardiac resynchronization therapy [42] and not simply PVR, may be needed. Future studies which focus on patients with marked interventricular dyssynchrony who undergo PVR are needed to definitely address this proposition.

### Limitations

Several limitations of this study are worth noting. Assessments were performed at a mean of 7 months following PVR. Available data suggests that RV volumes and, thus, EF remain stable between 1 and 5 years after PVR [43]. Nevertheless, we cannot exclude the possibility that changes in strain and synchrony might develop with longer follow-up even in the absence of prosthetic valve dysfunction. The distal RV outflow tract wall was not analyzed because in many patients it consisted of thin patch material from the initial TOF repair and was not amenable to feature tracking analysis. The temporal resolution of the cine CMR data was relatively low; consequently, small changes in synchrony parameters may have gone undetected. However, as the temporal resolution for each patient was similar on the pre- and post-PVR CMR studies, the synchrony changes which were identified are likely valid. The small sample size of the study may have limited the ability to detect small changes in strain and synchrony parameters. Similarly, the power of our study was not sufficient to assess the influence of age at initial repair and age at PVR on the impact of PVR. Finally, a comparison between the PVR alone versus the PVR with RV remodeling cohorts was not reported owing to the small numbers in each subgroup. We acknowledge that RV remodeling may have some impact on regional mechanics, but our study was not adequately powered to address this question.

## Conclusions

In this cohort, surgical PVR in repaired TOF patients resulted in improved LV global strain and no change in RV global strain. LV synchrony parameters improved or were unchanged, RV synchrony effects varied by location, and interventricular synchrony was largely unchanged. Future studies should address longer-term changes in strain and synchrony following PVR and their relationship to clinical outcomes.

## Abbreviations

CCD: cross-correlation delay
CMR: cardiovascular magnetic resonance
EDV: end-diastolic volume
EF: ejection fraction
ESV: end-systolic volume
ICC: intraclass correlation coefficient
LV: left ventricle/left ventricular
PR: pulmonary regurgitation
PVR: pulmonary valve replacement
RV: right ventricle/right ventricular
TOF: Tetralogy of Fallot
